# The role of cytoreductive surgery in multifocal/multicentric glioblastomas

**DOI:** 10.1007/s11060-023-04410-7

**Published:** 2023-09-12

**Authors:** Diyan Dimov, Daniel Brainman, Björn Berger, Roland Coras, Alexander Grote, Matthias Simon

**Affiliations:** 1 Department of Neurosurgery, Evangelisches Klinikum Bethel, Universitätsklinikum OWL, Bielefeld, Germany; 2 Department of Neuroradiology, Evangelisches Klinikum Bethel, Universitätsklinikum OWL, Bielefeld, Germany; 3 Department of Neuropathology, Erlangen University Hospital, Friedrich-Alexander University Erlangen-Nürnberg, Erlangen, Germany; 4 Present Address: Department of Neurosurgery, Universitätsklinikum Giessen und Marburg, Marburg, Germany

**Keywords:** Multifocal, Multicentric, Glioblastoma, Resection, Biopsy, Growth patterns

## Abstract

**Purpose:**

Multifocal/multicentric glioblastomas (mGBM) account for up to 20% of all newly diagnosed glioblastomas. The present study investigates the impact of cytoreductive surgery on survival and functional outcomes in patients with mGBM.

**Methods:**

We retrospectively reviewed clinical and imaging data of 71 patients with newly diagnosed primary (IDH1 wildtype) mGBM who underwent operative treatment in 2015–2020 at the authors’ institution. Multicentric/multifocal growth was defined by the presence of ≥ 2 contrast enhancing lesions ≥ 1 cm apart from each other.

**Results:**

36 (50.7%) patients had a resection and 35 (49.3%) a biopsy procedure. MGMT status, age, preoperative KPI and NANO scores as well as the postoperative KPI and NANO scores did not differ significantly between resected and biopsied cases. Median overall survival was 6.4 months and varied significantly with the extent of resection (complete resection of contrast enhancing tumor: 13.6, STR: 6.4, biopsy: 3.4 months; P = 0.043). 21 (58.3%) of resected vs. only 12 (34.3%) of biopsied cases had radiochemotherapy (p = 0.022). Multivariate analysis revealed chemo- and radiotherapy and also (albeit with smaller hazard ratios) extent of resection (resection vs. biopsy) and multicentric growth as independent predictors of patient survival. Involvement of eleoquent brain regions, as well as neurodeficit rates and functional outcomes did not vary significantly between the biopsy and the resection cohorts.

**Conclusion:**

Resective surgery in mGBM is associated with better survival. This benefit seems to relate prominently to an increased number of patients being able to tolerate effective adjuvant therapies after tumor resections. In addition, cytoreductive surgery may have a survival impact per se.

**Supplementary Information:**

The online version contains supplementary material available at 10.1007/s11060-023-04410-7.

## Introduction

Glioblastoma has been conceptualized as a systemic brain disease with a somewhat circumscribed beginning, which therefore can often be successfully treated initially with local measures such as surgery and radiotherapy [1]. However, approximately 20% of cases already present with multifocal and/or multicentric disease (mGBM) [2–8]. Multifocal glioblastoma is usually defined by MR imaging showing several contrast enhancing lesions connected by FLAIR hyperintense signal thought to represent tumor infiltration, i.e. migrating tumor cells (as opposed to multicentric disease in which these FLAIR bridges are absent) [2, 3, 5, 9–11].

Current neuro-oncological therapies rest heavily on a tissue and even molecular diagnosis. Hence, obtaining some tissue is mandatory in all glioblastoma cases including patients with multifocal/multicentric disease. The role of additional resective surgery for a circumscribed glioblastomas is well established [12, 13]. However, in everyday clinical practice also many cases with mGBM undergo cytoreductive surgery. This is usually based on the assumption that the traditional “all or nothing” rationale for glioblastoma surgery is an improper simplification of a more complex relation [12–18]. Patients are believed to derive some benefit already from a subtotal tumor removal even if these effects are smaller than the survival impact of a complete resection. There are important technical challenges and restrictions. Extensive resections are usually quite difficult or even impossible to achieve when one is confronted with multiple lesions in different parts of the brain.

In view of these issues we have analyzed our recent institutional experience with the surgical management of patients with mGBM. To this end, we compared patient survival following resective vs. bioptic surgery. We also studied various growth and spread patterns, as well as clinical parameters as possible prognostic predictors, and we assessed functional outcomes.

## Patients and methods

### Patients and clinical data

We identified 434 patients > 18 years of age undergoing their first surgery in our department for a histologically confirmed glioblastoma from January 2015 to December 2020 in our institutional database. Preoperative imaging data and radiological reports were reviewed and patients were included in the present study if they were found to harbor a multifocal or multicentric tumor (for criteria and radiological data, please see below), and if the neuropathological studies diagnosed a IDH-wildtype glioblastoma, i.e. if at least immunohistochemical studies had been performed showing no expression of mutant IDH1. The final study cohort comprised 71 cases.

Clinical data were collected retrospectively from the patients’ charts. If required patients were also contacted by phone. Progression was defined as institution of a new oncological treatment or of palliative care. Functional outcomes were assessed using the postsurgical (discharge) KPI and NANO scores [19], and the occurrence of surgical, non-temporary neurological or medical complications. The severity of complications was graded using the CTCAE scheme [20] and neurological complications were considered temporary if they resolved within 30 day [21].

### Surgical treatment

All cases were discussed in our interdisciplinary neuro-oncological tumor conference. Throughout the study period we offered diagnostic surgery to all patients with a presumed mGBM if patient age and clinical performance status appeared to allow for adjuvant therapy following operative treatment. A tumor resection rather than a biopsy was recommended for large and symptomatic lesions on an individual basis. Open navigation-guided microsurgical rather than stereotactic biopsies were performed for selected non-eloquent and superficially located lesions. A robotic system (neuromate®, Renishaw GmbH, Pliezhausen, Germany) was employed for stereotactic bioptic surgery. We have recently published the technical details and a critical evaluation of the procedure [22]. Surgical adjuncts such as ALA fluorescence, neuromonitoring and awake craniotomies were used for resective surgery as required by lesion location and extension, and deemed useful and/or necessary by the operating surgeon.

### Radiological data review volumetry

**Fig. 1:**
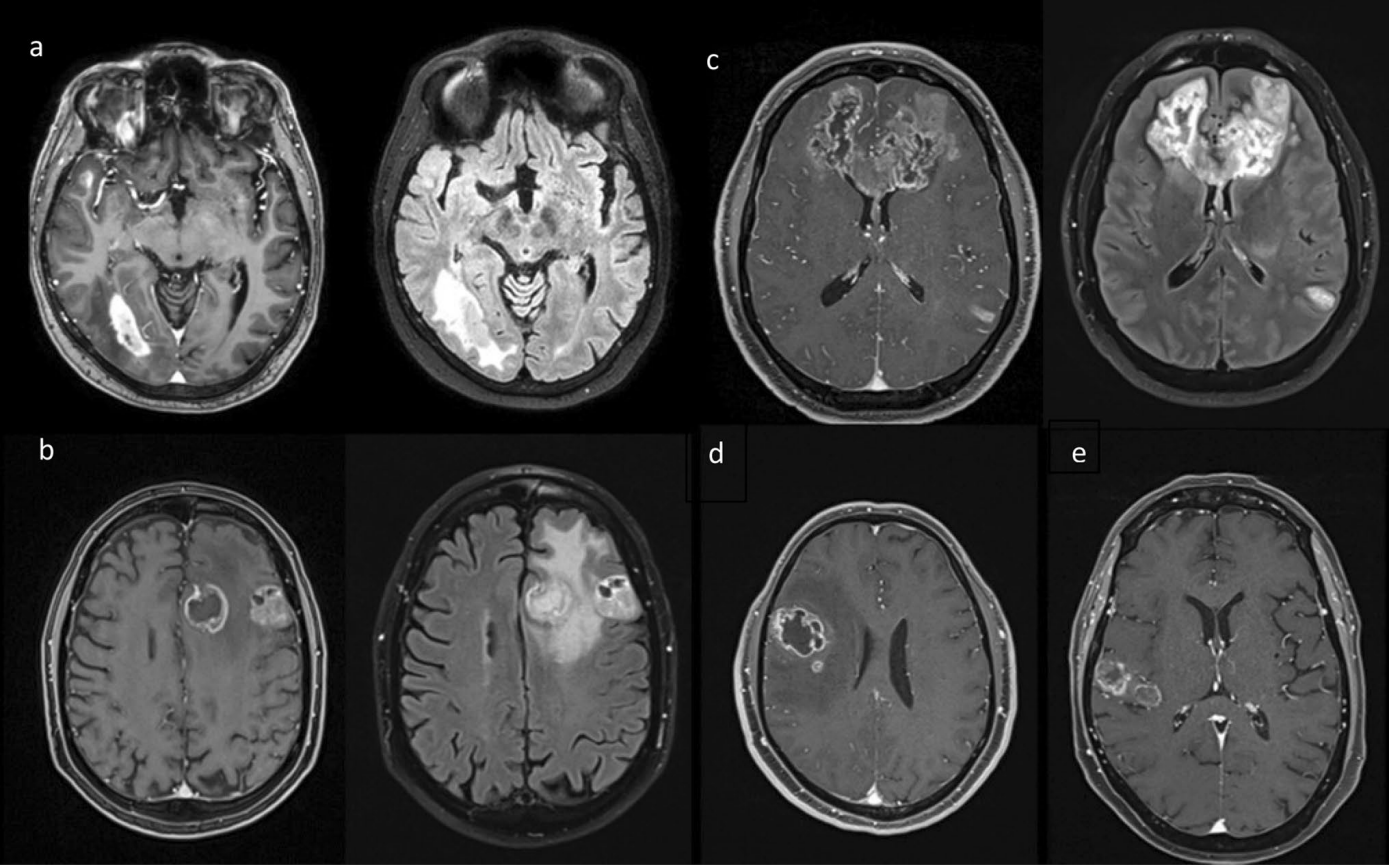
Radiological characteristics of multicentric and multifocal growth patterns. **a** T1 with contrast and FLAIR weighted MR scans showing two contrast enhancing lesions separated by > 1 cm without a T2/FLAIR bridge—multicentric growth pattern. **b** T1 and FLAIR weighted images with two contrast enhancing lesions separated by > 1 cm connected by a FLAIR hyperintense signal—multifocal growth pattern. **c** T1 and FLAIR weighted scans depicting three contrast enhancing foci (and possibly a fourth FLAIR hyperintense lesion in the left thalamus). There is a FLAIR hyperintense signal connecting the frontal foci, but no such bridge between the left temporodorsal tumor manifestation and the other lesions—simultaneous multicentric and multifocal growth pattern. **d** T1 weighted scan with a smaller contrast enhancing lesion located within 1 cm of the main lesion—unifocal growth pattern with satellite lesion. **e** T1 weighted image showing two contrast enhancing lesions of similar size within 1 cm of one another—unifocal growth pattern

The preoperative MRI studies from all 71 cases were subjected to a neuroradiological review. The designation mGBM required the presence of two or more contrast-enhancing lesions separated by > 1 cm. Cases with only a “perilesional” or “satellite” growth pattern with several discrete lesions but < 1 cm apart from each other were excluded from our analysis [2, 3, 7]. A FLAIR hyperintense signal connecting two lesions defined a multifocal growth pattern, and the lack thereof multicentric spread. Hence, a case with three or more lesions could be categorized as showing both multifocal and multicentric growth if only some of the foci were found to be joined by FLAIR hyperintense tissue (Fig. 1). We also documented bihemispheral, periventricular (< 1 cm distance of a contrast enhancing mass from the ventricle) and pericallosal growth (contrast enhancing tumor within the corpus callosum or within 1 cm from its lateral edge defined by the superolateral border of the lateral ventricle), as well as subarachnoid or subependymal spread (Supplementary Fig. 1) [2, 7].

In order to evaluate the respective patient’s tumorload, we counted contrast enhancing measurable lesions > 1 cm following the RANO criteria [23] and used computer-assisted volumetric analyses of the contrast enhancing tissues employing a well-established computer software (iplanNet, Brainlab AG, Munich, Germany). Location was assessed as involvement of one or more of the following regions: frontal, temporal, parietal and occipital lobe, the cerebellum, brainstem and/or deep midline structures (insula, basal ganglia, thalamus, hypothalamus, internal capsule). All measurable lesions were scored for possible eloquence using the three-tiered scheme originally described by Sawaya et al. [24] and also separately for motor, speech and/or visual pathways eloquence [25]. Cases were assigned to the respective eloquence categories based on the most eloquent tumor manifestation.

### Statistical analysis

Routine statistical analyses were performed using IBM SPSS Statistics for Windows (Version 25.0, IBM Corp., Armonk, NY). Two-sided tests were employed throughout. We considered p values < 0.05 as significant. Survival was studied with Kaplan Meier estimates. We employed Cox regression modelling (inclusion procedure) for multivariate survival analyses.

## Results

### Patient demographics and clinical presentation

We analyzed a total of N = 71 patients (Table 1). Clinical presentation included a reduced KPI ≤ 70 in 23 (32.4%), a NANO score of 3 or more in 36 (50.7%), and seizures in 25 (35.2%) patients.

### Tumor growth patterns and spread

The preoperative MR imaging studies showed multifocal disease in 54 (76.1%) cases and multicentric tumor growth in 26 (36.6%; both: N = 9, 12.7%) (Fig. 1). Bilateral contrast enhancing lesions were seen in 37 (52.1%) patients. Twenty-six (36.6%) patients had ≥ 3 discrete and measurable lesions, and in nine patients the MR scans showed non-measurable disease. Forty-one patients presented with eloquent (57.7%), and 21 cases (29.6%) with near-eloquent tumor manifestations. Motor, language and visual pathway eloquent lesions were seen in 24 (33.8%), 12 (16.9%) and 26 (36.6%) of patients (Table 1).

**Table 1:** Demographics, radiological and treatment data

	Characteristics		Value
Demographics	Age (years)	Mean ± SD (yrs.)	66.7 ± 13.3
		Median (25–75% IQR, yrs.)	69.0 (58.0–78.0)
	Sex	Male	36 (50.7%)
		Female	35 (49.3%)
	Preoperative KPI	Mean ± SD	77.5 ± 15.7
		Median (25–75% IQR)	80 (70–90)
	Preoperative NANO score	Mean ± SD	2.85 ± 2.29
		Median (25–75% IQR)	3 (1–4)
	Postoperative KPI	Mean ± SD Median (25–75% IQR)	77.0 ± 17.7 80 (70–90)
	Postoperative NANO	Mean ± SD Median (25–75% IQR)	2.89 ± 2.47 2 (1–4)
	Preoperative seizures	Yes	25 (35.2%)
		No	46 (64.8%)
	MGMT promoter hypermethylation^a^	Yes	27 (38.0%)
		No	34 (47.9%)
Radiological data			
Growth pattern	Multicentric	Yes	26 (36.6%)
		No	45 (63.4%)
	Multifocal	Yes	54 (76.1%)
		No	17 (23.9%)
Spread	Bilateral	Yes	37 (52.1%)
		No	34 (47.9%)
	Periventricular	Yes	64 (90.1%)
		No	7 (9.9%)
	Pericallosal	Yes	53 (74.6%)
		No	18 (25.4%)
	Subarachnoid	Yes	12 (16.9%)
		No	59 (83.1%)
	Subependymal	Yes	12 (16.9%)
		No	59 (16.9%)
	“butterfly”^b^	Yes	35 (49.3%)
		No	36 (50.7%)
Tumor localization	Frontal	Yes	51 (71.8%)
		No	20 (28.2%)
	Parietal	Yes	44 (62.0%)
		No	27 (38.0%)
	Temporal	Yes	34 (47.9%)
		No	37 (52.1%)
	Occipital	Yes	17 (23.9%)
		No	54 (76.1%)
	Insula, basal ganglia, (hypo)thalamus	Yes	33 (46.5%)
		No	38 (53.5%)
	Cerebellum	Yes	5 (7.0%)
		No	66 (93.0%)
	Brainstem	Yes	4 (5.6%)
		No	67 (94.4%)
Eloquence	Overall	≥ 1 eloquent lesion	41 (57.7%)
		≥ 1 near-eloquent, but no eloquent lesion	21 (29.6%)
		No (near) eloquent lesion(s)	9 (12.7%)
	≥ 1 any motor eloquent lesion	Yes	24 (33.8%)
		No	47 (66.2%)
	≥ 1 speech eloquent lesion	Yes	12 (16.9%)
		No	59 (83.1%)
	≥ 1 visual pathways eloquent lesion	Yes	26 (36.6%)
		No	45 (63.4%)
Tumorload	Lesion no.	Mean ± SD Median (25–75% IQR)	2.94 ± 2.10 2 (1–3)
	Preoperative tumorload (volume)	Mean ± SD Median (25–75% IQR, ml)	23.3 ± 20.5 17.3 (8.1–31.9)
	Postoperative tumorload (volume)	Mean ± SD (ml) Median (25–75% IQR, ml)	10.6 ± 15.0 4.5 (0.3–15.0)
Treatment data			
Operative treatment	Type of operation	Complete resection (postop. contrast enhancing tumor volume < 0.1 ml)	13 (18.3%)
		STR	23 (32.4%)
		Biopsy	35 (49.3%)
	Extent of resection	Mean ± SD Median (25–75% IQR, %)	45.1 ± 46.9% 25.0 (0-98.4)
Adjuvant therapy	Radiotherapy	Completed	38 (53.5%)
		Incomplete	10 (14.1%)
		None	23 (32.4%)
	Chemotherapy	Yes	40 (56.3%)
		No	31 (43.7%)
	Radiochemotherapy	RT completed, TMZ or TMZ/CCNU	33 (46.5%)
		RT incomplete or monotherapy	16 (22.5%)
		None	22 (31.0%)

^a^N = 61

^b^“butterfly” = bihemspheric contrast enhancing pericallosal disease

*SD* standard deviation,* IQR* interquartile range, *KPI* Karnofsky performance index, *NANO* neurologic assessment in neuro-oncology, *MGMT* O(6)-methylguanine-DNA methyltransferase, *STR* subtotal resection, *RT* Radiotherapy, *TMZ* Temozolomide, *CCNU* Lomustine

### Surgical management and functional outcomes

Thirty-six patients (50.7%) had tumor resections. In 14 cases we aimed at a complete resection of two (13) or three (1) contrast enhancing tumors during the same surgery (unilateral disease: 11; bifrontal paramedian disease: 3). The remaining 22 patients (uni-/bilateral disease: 10/12, > 2 lesions: 6) had a resection of the largest lesion only, i.e. a planned subtotal resection of the contrast enhancing tissues. The overall mean extent of resection was 45.1 ± 46.9% (median: 25.0, IQR: 0-98.4%), but 88.9 ± 19.8% (median 98.2, IQR: 87.4–100%) in the resective cohort. This includes 13 (36.1%) cases with a complete resection (defined by < 0.1 ml contrast enhancing signal in the early postoperative MR study). Four patients (5.6%) underwent an open microsurgical biopsy and stereotactic (roboter-guided) biopsies were performed in 31 cases (43.7%).

Four cases incurred CTCAE grades 3–5 new or worsened postoperative neurological deficits persisiting ≥ 30 days (5.6%; resection: N = 3, biopsy: N = 1), and five patients CTCAE grades 3–5 local/surgical complications (7.0%; resection: N = 3, biopsy: N = 2; including one brain abscess 3 months after surgery for temporal lobe glioblastoma and three hemorrhages). One case with a VP shunt implanted for normal pressure hydrocephalus required an operative shunt revision for shunt malfunction 13 days after a stereotactic biopsy. 30 days mortality was 4/71 (5.6%) with one patient dying from gastrointestinal bleeding 11 days following surgery, two from progressive tumors, and one from unknown causes. Median preoperative KPI and NANO scores were 80 (25–75% IQR: 20–100) and 3 (25–75% IQR: 1–4); the respective postoperative figures were 80 (25–75% IQR: 20–100) and 2 (25–75% IQR: 1–4). Only two cases (2.8%) had a postoperative ≥ 20 drop of their KPI score, and all neurologically intact patients retained their preoperative NANO score of 0.

### Follow-up, adjuvant treatment and survival outcomes

Median follow-up was 5.2 (mean: 7.8 ± 8.0) months with 62 patients (87.3%) followed until death. Postoperative radiotherapy was started in 48 (67.6%) and completed in 38 cases (53.5%). Forty patients (56.3%) had chemotherapy (all temozolomide, including three cases with CCNU/temozolomide combination chemotherapy [26]), and 33 (46.5%) had radiochemotherapy. Radiotherapy only was administered in 9 (12.7%) patients. Median overall survival was 6.4 (95% CI: 4.2–8.5) months, and median progression free survival was 4.1 (95% CI: 3.0-5.2) months.

Of note, the frequency and intensity of adjuvant treatment varied markedly between cases undergoing cytoreductive vs. bioptic surgery. E.g. 24/36 (66.7%) cases completed their course of radiotherapy following cytoreductive surgery vs. only 14/35 (40.0%; P = 0.028) after a biopsy procedure. Radiochemotherapy [26, 27] was given in 21/36 (58.3%) resective cases but only in 12/35 (34.3%; *P* = 0.022) of biopsy patients (Table 2).

**Table 2:** Demographics, radiological and treatment characteristics in the resection vs. biopsy cohorts

	Characteristics		Resection	Biopsy	P
Demographics	Age	≤ 69 yrs. (median)	20 (54.1%)	17 (45.9%)	NS
		> 69 yrs.	16 (47.1%)	18 (52.9%)	
	Sex	Female	17 (48.6%)	18 (51.4%)	NS
		Male	19 (52.8%)	17 (47.2%)	
	Preoperative KPI	80–100	26 (54.2%)	22 (45.8%)	NS
		< 80	10 (43.5%)	13 (56.5%)	
	Postoperative KPI	80–100	26 (54.2%)	22 (45.8%)	NS
		< 80	10 (43.5%)	13 (56.5%)	
	Preoperative NANO score	0–2	14 (40.0%)	21 (60.0%)	NS^b^
		> 2	22 (61.1%)	14 (38.9%)	
	Postoperative NANO score	0–2	20 (57.1%)	15 (42.9%)	NS
		> 2	16 (44.4%)	20 (55.6%)	
	Preoperative seizures	Yes	15 (60.0%)	10 (40.0%)	NS
		No	21 (45.7%)	25 (54.3%)	
	MGMT promoter hypermethylation^a^	Positive	16 (59.3%)	11 (40.7%)	NS
		Negative	15 (44.1%)	19 (55.9%)	
Radiological data					
Growth pattern	Multicentric	Yes	13 (50.0%)	13 (50.0%)	NS
		No	23 (51.1%)	22 (48.9%)	
	Multifocal	Yes	27 (50.0%)	27 (50.0%)	NS
		No	9 (52.9%)	8 (47.1%)	
Spread	Bilateral	Yes	15 (40.5%)	22 (59.5%)	NS^c^
		No	21 (61.8%)	13 (38.2%)	
	Periventricular	Yes	31 (48.4%)	33 (51.6%)	NS
		No	5 (71.4%)	2 (28.6%)	
	Pericallosal	Yes	26 (49.1%)	27 (50.9%)	NS
		No	10 (55.6%)	8 (44.4%)	
	Subarachnoid	Yes	4 (33.3%)	8 (66.6%)	NS
		No	32 (54.2%	27 (45.8%)	
	Subependymal	Yes	7 (58.3%)	5 (41.7%)	NS
		No	29 (49.2%)	30 (50.8%)	
	“butterfly”^d^	Yes	15 (42.9%)	20 (57.1%)	NS
		No	21 (58.3%)	15 (41.7%)	
Tumor localization		Lobar only	21 (58.3%)	15 (41.7%)	NS
		Any posterior fossa disease	2 (40.0%)	3 (60.0%)	
		Other	13 (43.3%)	17 (56.7%)	
Eloquence	Overall	≥ 1 eloquent lesion	23 (56.1%)	18 (43.9%)	NS
		≥ 1 near-eloquent, but no eloquent lesion	6 (28.6%)	15 (71.4%)	
		No (near) eloquent lesion(s)	7 (77.7%)	2 (22.2%)	
	≥ 1 any motor eloquent lesion	Yes	10 (41.7%)	14 (58.3%)	NS
		No	26 (55.3%)	21 (44.7%)	
	≥ 1 speech eloquent lesion	Yes	7 (58.3%)	5 (41.7%)	NS
		No	29 (49.2%)	30 (50.8%)	
	≥ 1 visual pathways eloquent lesion	Yes	13 (50.0%)	13 (50.0%)	NS
		No	23 (51.1%)	22 (48.9%)	
Tumorload	Lesion no.	≤ 2 (median)	29 (64.4%)	16 (35.6%)	0.002
		> 2	7 (26.9%)	19 (73.1%)	
	Preoperative	> 17.3 ml (median)	22 (62.9%)	13 (37.1%)	0.043
		≤ 17.3 ml	14 (38.9%)	22 (61.1%)	
	Postoperative	> 4.5 ml (median)	7 (20.0%)	28 (80.0%)	< 0.001
		≤ 4.5 ml	29 (80.6%)	7 (20.0%)	
Treatment data					
Adjuvant therapy	Radiotherapy	Completed	24 (63.2%)	14 (36.8%)	0.028
		Incomplete	4 (40.0%)	6 (60.0%)	
		None	8 (34.8%)	15 (65.2%)	
	Chemotherapy	Yes	26 (65.0%)	14 (35.0%)	0.006
		No	10 (32.3%)	21 (67.7%)	
	Radiochemotherapy	RT completed, TMZ or TMZ/CCNU	21 (63.6%)	12 (36.4%)	0.022
		RT incomplete or monotherapy	8 (50.0%)	8 (50.0%)	
		None	7 (31.8%)	15 (68.2%)	

^a^N = 61

^b^P = 0.075

^c^P = 0.074

^d^“butterfly” = bihemspheric contrast enhancing pericallosal disease

*KPI* Karnofsky performance index, *NANO* eurologic assessment in neuro-oncology, *MGMT* O(6)-methylguanine-DNA methyltransferase, *STR* subtotal resection, *RT* Radiotherapy, *TMZ* Temozolomide, *CCNU* Lomustine

**Fig. 2:**
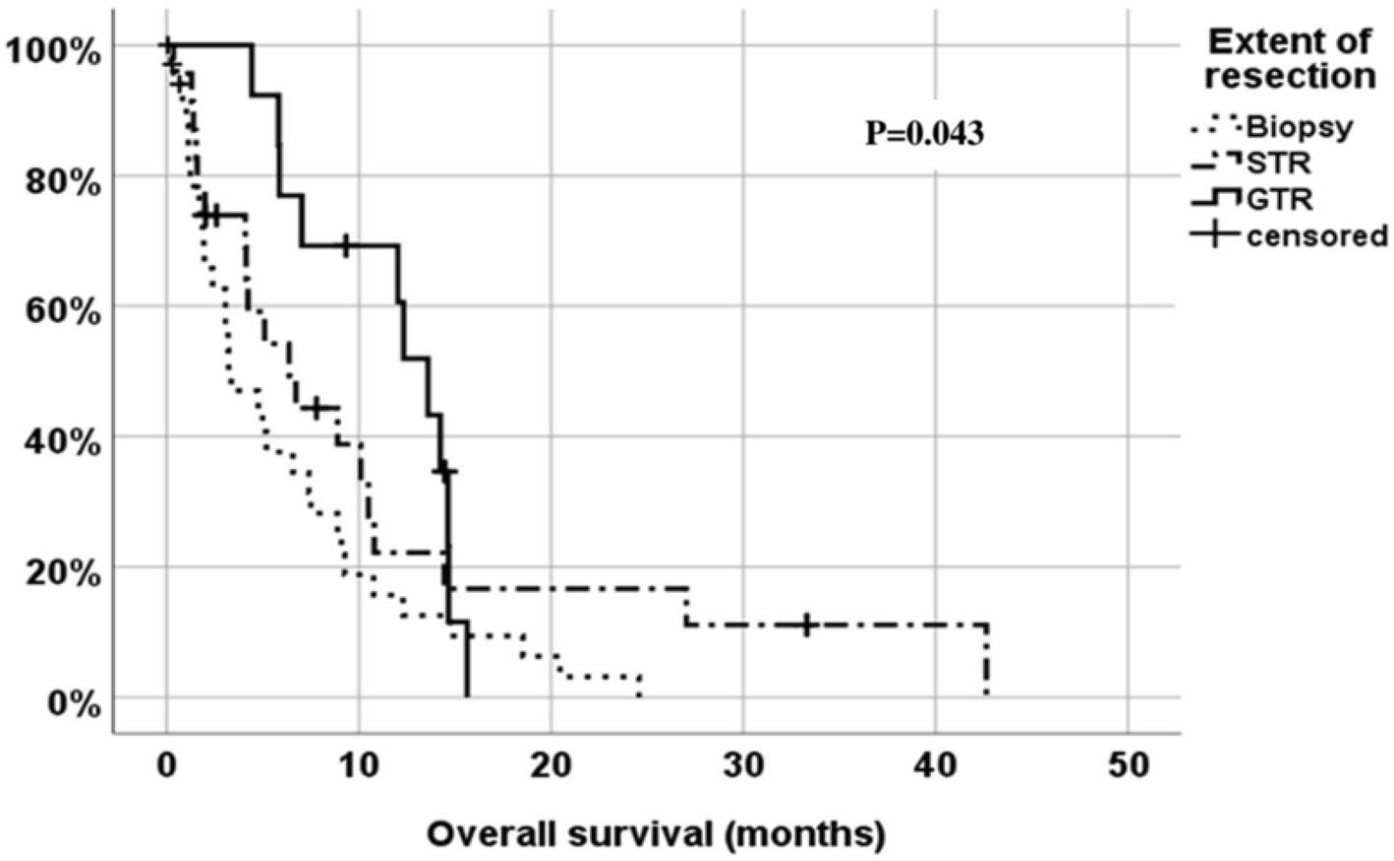
Prognostic impact of the extent of resection on overall survival (Kaplan-Meier analysis), *STR* subtotal resection, Complete res. – postop. contrast enhancing tumor volume < 0.1 ml

Treatment related variables had a strong survival impact (Table 3). Median estimated survival was 10.8 months in patients who completed radiotherapy and had chemotherapy, but only 1.5 months in cases with no adjuvant therapy. There was a statistically significant correlation between extent of resection and survival (Fig. 2). Median survival was 13.6 (95% CI: 11.1–16.1) months after a complete resection (of the contrast enhancing tumor), 6.4 (95% CI: 2.8–10.0) months after a subtotal resection (STR), and only 3.4 (95% CI: 1.05.7) months after a biopsy (P = 0.043). Median survival was 12.0 (95% CI: 7.2–16.8) months following a resection and radiochemotherapy. Finally, multicentric tumor growth was associated with a significantly worsened survival.

**Table 3:** Prognostic significance of demographics, radiological and treatment data

	Characteristics		N	mOS	95% CI	P
Demographics	Age	≤ 69 yrs.	37	9.1	6.0–12.2	0.008
		> 69 yrs. (median)	34	3.1	0.5–5.7	
	Sex	Female	35	6.6	2.9–7.5	NS
		Male	36	5.2	3.1–10.0	
	Preoperative KPI	80–100	48	8.9	5.6–12.2	NS^b^
		< 80	23	3.1	1.3–4.8	
	Postoperative KPI	80–100	48	8.9	5.6–12.2	NS^c^
		< 80%	23	3.1	1.2–4.9	
	Preoperative NANO score	0–2	35	6.4	4.1–8.7	NS
		> 2	36	5.8	0.2–11.4	
	Postoperative NANO score	0–2	36	7.0	5.2–8.9	NS
		> 2	35	4.8	3.3–6.3	
	Preoperative seizures	Yes	25	7.0	2.0–12.0	NS
		No	46	5.8	4.1–7.6	
	MGMT promoter hypermethylation^a^	Positive	27	4.2	1.8–6.7	NS
		Negative	34	7.0	4.8–9.3	
Radiological data						
Growth pattern	Multicentric	Yes	26	3.2	5.7–12.1	0.019
		No	45	8.9	0.5–5.9	
	Multifocal	Yes	54	6.6	3.0–5.8	NS
		No	17	4.4	4.8–8.4	
Spread	Bilateral	Yes	37	6.6	2.7–10.5	NS
		No	34	5.9	3.7–8.1	
	Periventricular	Yes	64	5.2	2.9–7.5	NS
		No	7	9.3	3.5–15.0	
	Pericallosal	Yes	53	5.8	3.2–8.5	NS
		No	18	7.0	0–14.2	
	Subarachnoid	Yes	12	6.7	0.9–12.6	NS
		No	59	5.9	3.6–8.2	
	Subependymal	Yes	12	2.4	0–6.1	NS
		No	59	7.0	3.7–10.3	
	„butterfly“^e^	Yes	35	7.4	2.7–12.1	NS
		No	36	5.9	4.0–7.7	
Tumor localization		Lobar only	36	8.9	5.4–12.4	NS
		Any posterior fossa disease	5	1.5	0.7–2.4	
		Other	30	5.0	3.9–6.1	
Eloquence	Overall	> 1 eloquent lesion	41	5.9	3.6–8.1	NS
		> 1 near-eloquent, but no eloquent lesion	21	7.4	0–15.9	
		No (near) eloquent lesion(s)	9	6.4	4.8–7.9	
	Motor eloquence	Yes	24	4.8	4.5–10.3	NS
		No	47	7.4	3.3–6.2	
	Speech eloquence	Yes	12	4.1	1.9–6.3	0.022
		No	59	7.0	3.9–10.1	
	Visual pathways eloquence	Yes	26	5.2	2.2–8.2	NS
		No	45	6.6	3.7–9.4	
Tumorload	Lesion no	≤ 2 (median)	45	8.9	4.4–13.4	NS^d^
		> 2	26	3.1	1.1–5.0	
	Preoperative	> 17.3 ml (median)	35	4.2	2.9–5.6	NS
		≤ 17.3 ml	36	8.9	6.0–11.8	
	Postoperative	> 4.5 ml (median)	35	3.4	0.7–6.1	NS
		≤ 4.5 ml (median)	36	7.0	3.3–10.8	
Treatment data						
Operative treatment	Type of operation	Resection	36	10.1	5.1–15.1	0.015
		Biopsy	35	3.4	1.0–5.7	
	Extent of resection	Complete resection (postop. contrast enhancing tumor volume < 0.1 ml)	13	13.6	11.1–16.1	0.043
		STR	23	6.4	2.8–10.0	
		Biopsy	35	3.4	1.0–5.7	
Adjuvant therapy	Radiotherapy (RT)	Completed	39	10.2	7.6–12.6	< 0.001
		Incomplete	9	5.9	2.2–9.5	
		None	23	1.5	1.1–2.0	
	Chemotherapy (CT)	Yes	40	10.5	8.3–12.7	< 0.001
		No	31	1.9	1.5–2.4	
	Radiochemotherapy	RT completed, TMZ or TMZ/CCNU	33	10.8	7.9–13.7	< 0.001
		RT incomplete or monotherapy	16	5.9	0.9–4.0	
		None	22	1.5	0.2–1.0	

^a^N = 61

^b^P = 0.066

^c^P = 0.067

^d^P = 0.065

^e^“butterfly” = bihemspheric contrast enhancing pericallosal disease

*mOS* median overall survival (months); 95%CI – 95% confidence interval, *MGMT* O(6)-methylguanine-DNA methyltransferase, *KPI* Karnofsky performance index, *NANO* neurologic assessment in neuro-oncology, *STR* subtotal resection, *RT* Radiotherapy, *TMZ* Temozolomide, *CCNU* Lomustine

Four cases incurred CTCAE grades 3–5 new or worsened postoperative neurological deficits persisiting ≥ 30 days (5.6%; resection: N = 3, biopsy: N = 1), and 5 cases with CTCAE grades 3–5 local/surgical complications (7.0%; resection: N = 3, biopsy: N = 2; including one brain abscess 3 months after surgery for temporal lobe glioblastoma and three hemorrhages). One case with a VP shunt implanted for normal pressure hydrocephalus required an operative shunt revision for shunt malfunction 13 days after a stereotactic biopsy. 30 days mortality was 4/71 (5.6%) with one patient dying from gastrointestinal bleeding 11 days following surgery, two from progressive tumors, and one from unknown causes. Median preoperative KPI and NANO scores were 80 (25–75% IQR: 20–100) and 3 (25–75% IQR: 1–4); the respective postoperative figures were 80 (25–75% IQR: 20–100) and 2 (25–75% IQR: 1–4). Only two cases (2.8%) had a postoperative > 20 drop of their KPI score, and all neurologically intact patients retained their preoperative NANO score of 0.

A multivariate Cox regression analysis (Supplementary Table 1) revealed multicentric growth, biopsy vs. resective surgery, no chemotherapy and no or incomplete radiotherapy as independent negative prognostic predictors with the largest hazard ratios attributed to the adjuvant therapy variables.

### Surgical treatment bias

We extensively compared the resection and biopsy patients with respect to demographic factors as well as tumor characteristics (Table 2). Neither age, sex, MGMT status nor preoperative KPI or NANO scores varied significantly between biopsy and resection cases. There was a statistical trend for an association between cytoreductive surgery and a worse preoperative neurological condition as assessed by the NANO score. Cases with three or more lesions had significantly more often bioptic than resective surgery. However, the volumetric tumorload was significantly higher in resective vs. biopsy cases. There was a statistical trend in favor of performing a biopsy in patients with bihemispheral tumor growth.

## Discussion

The optimal management of patients with mGBM is controversial [1–6]. Our data suggest that resective surgery in cases with mGBM might be beneficial, and that much of this effect relates to the impact of tumor debulking and reduction of mass effect which allows the patient to undergo adjuvant therapies. However, we also found some evidence that surgical cytoreduction per se might prolong patient survival.

Patient survival following the diagnosis of a mGBM is generally poor. In the literature overall survival varies between 3 and 9 months [2–9]. Median overall survival in the present series was only 6.4 months. However, there was significant interindividual variation. Median survival after a complete resection of the contrast enhancing tumor (which was achieved in 13 [18.3%] cases) was 13.6 months. While the patients’ prognosis was found to correlate significantly also with traditional prognostic factors such as age and KPI, treatment variables appeared to play an even more prominent role. Based on the hazard ratios in the multivariate analysis completion of postoperative adjuvant radiotherapy and chemotherapy were the strongest positive predictors of patient survival. Interestingly, there was a strong correlation between more and completed adjuvant therapy with resective rather than bioptic surgery. This may indicate that successful adjuvant therapy often requires upfront debulking surgery because patients with a large tumorload will not tolerate these treatments because of the mass effect of the tumor [3, 10].

The risk of incurring a neurological deficit and/or complications in general is of course a major concern in cases with limited survival which in addition need active postoperative oncological therapy in order to realize the benefits of their surgery. Surgical management of our patients carried a quite significant but probably still acceptable complication rate. We observed 5.6% new or worsened CTCAE grade 3–5 neurological deficits ≥ 30 days and 7.0% CTCAE grade 3–5 local/surgical complications. Figures were larger for resective than bioptic cases, but the overall small numbers precluded any statistical significance. Still, the well-known lower complication rate of (stereotactic) biopsies when compared to open microsurgery is a relevant issue when dealing with a patient population with a very limited survival prognosis [21]. Nevertheless, at least in this series, the use of resective surgery for mGBM did not result in a relevant number of patients incurring deficits and complications precluding further therapy and thereby shortening survival.

Interestingly, our data and especially the multivariate analysis also suggest that surgical cytoreduction as such may have a significant impact on patient survival. These findings are well in line with the results detailed in the recent studies by Di et al. and Friso et al. The existence of a correlation between degree of resection and survival also in mGBM should not come as a complete surprise. There is a considerable database suggesting that this relationship in (unifocal) GBM cannot be appropriately described by the all-or nothing-paradigm [11]. The extent of resection cut-off for a survival benefit derived from surgery may be in the range of 80–90% [14, 16, 17]. Interestingly (and quite fittingly), the mean extent of resection in cases from this study who had open debulking surgery was 88.9 ± 19.8%. In other words, at least in the present cohort a substantial number of cases had a resection of their contrast-enhancing tumor to a degree believed to be beneficial if performed for unifocal disease. If unifocal and mGBM respond similarly to surgical cytoreduction patients with mGBM could potentially be included in the same (surgical) clinical trials as cases with unifocal disease.

This line of reasoning does not take into account the issue of non-contrast enhancing glioblastoma tissues [15]. Against this background our finding that the presence of multicentric growth predicted a worse survival outcome might be of importance. Multicentric growth is defined by the absence of FLAIR/T2 hypertintense tissue bridges connecting contrast enhancing glioblastoma manifestations and might therefore somewhat resemble cancer metastasis. Multifocal growth on the other hand might be caused by infiltration and (mass cell) migration which will result in a larger and extensive but essentially still conceptually “unifocal” non contrast enhancing tumor. Resecting several contrast-enhancing foci in such cases can be conceptualized as multiple partial resections. Other groups have also compared different growth patterns and have not reported similar results [2–5, 8].

Finally, one of the key arguments against the existence of a causative relationship between degree of resection and survival is the presumed presence of surgical treatment bias, i.e. the notion that cases with an inherently better prognosis receive more aggressive therapy [2, 18, 28, 29]. We did not obtain evidence in favor of such bias with respect to established prognostic factors. We compared our resection and biopsy cohorts quite carefully. The patient subsets did not differ statistically significantly with respect to age, sex, functional (KPI) and neurological (NANO score) status, or preoperative seizure incidence. The rate of tumors with MGMT promoter hypermethylation was also quite similar. The cohorts only differed with respect to parameters describing tumor growth and spread. Tumors with two discrete lesions were much more likely to undergo resective surgery than a biopsy procedure, while higher volumetric tumorload was associated with a tumor resection. The general concept of maximal safe surgery of course precluded resecting some eloquent lesions and if possible resulted in choosing non-eloquent lesions as the biopsy target. This clearly constitutes the major treatment selection bias in this series.

### Limitations

Our study has of course significant shortcomings. The overall number of patients investigated was limited. A relatively high proportion of our cases had no or did not complete adjuvant radio- and chemotherapy. Data were retrieved only retrospectively. While surgical treatment followed an institutional protocol, this was not the case with the adjuvant therapies. Many patients were followed at outside institutions.

## Conclusions

We provide data to show that resective surgery somewhat counterintuitively may carry a survival benefit in cases with presumed mGBM glioblastoma. This benefit seems to relate prominently to an increased number of patients being able to tolerate effective adjuvant therapies after tumor resections, i.e. our results support the concept of operating in order to gain time and create space for chemo- and radiotherapy. In addition, cytoreductive surgery may have a survival impact per se. Surgical decision making in patients with mGBM should therefore focus on a proper balance between surgical risks, treating mass effect and—if possible—oncologically effective cytoreduction. This is actually very similar to current strategies for unifocal glioblastoma, i.e. also cases with mGBM should be considered for a tumor resection as long as an extensive and safe removal of contrast enhancing tissues is reasonably possible and patients are deemed to be able to undergo effective adjuvant chemo- and radiotherapy.

### Supplementary Information

Below is the link to the electronic supplementary material.

Supplementary material 1 (DOCX 188.1 kb)

## Data Availability

The datasets generated during and/or analysed during the current study are available from the corresponding author on reasonable request.

## Abbreviations

ALA: 5-aminolevulinic acid
CCNU: lomustine
CeTeG: lomustine-temozolamide radiochemotherapy combination
CI: Confidence interval
CTCAE: Common terminology criteria for adverse events
FLAIR: Fluid-attenuated inversion recovery
GBM: Glioblastoma
IDH1: Isocitrate dehydrogenase 1
IQR: Interquartile range
KPI: Karnofsky performance Index
MGMT: O(6)-methylguanine-DNA methyltransferase
MR: Magnetic resonance
mGBM: Multifocal/multicentric glioblastoma
NANO: Neurologic assessment in neuro-oncology
RANO: Response assessment in neuro-oncology
STR: Subtotal resection
SD: Standard deviation
TMZ: Temozolamide
VP: Ventriculoperitoneal
