# Supplementary material for: The role of cytoreductive surgery in multifocal/multicentric glioblastomas
Source: J Neurooncol. 2023 Sep 12;164(2):447–59. doi: 10.1007/s11060-023-04410-7 (PMC10522503; doi:10.1007/s11060-023-04410-7)
Supplement: Supplementary file 1 — Supplementary material 1 (DOCX 188.1 kb) [file 11060_2023_4410_MOESM1_ESM.docx]

Supplementary Table 1. Multivariate Cox Analysis of Overall Survival

| Characteristics | HR | 95% CI | P |
| --- | --- | --- | --- |
| Age (yrs.) | 1.01 | 0.99-1.03 | 0.426 |
| Multicentric growth | 1.95 | 1.09-3.48 | 0.024 |
| Resection vs. biopsy | 0.52 | 0.30-0.91 | 0.021 |
| Radiotherapy completed | 0.29 | 0.15-0.56 | <0.001 |
| Chemotherapy | 0.31 | 0.15-0.63 | 0.001 |
|  |  |  |  |

HR – Hazard Ratio; 95%CI – 95% confidence Interval

**
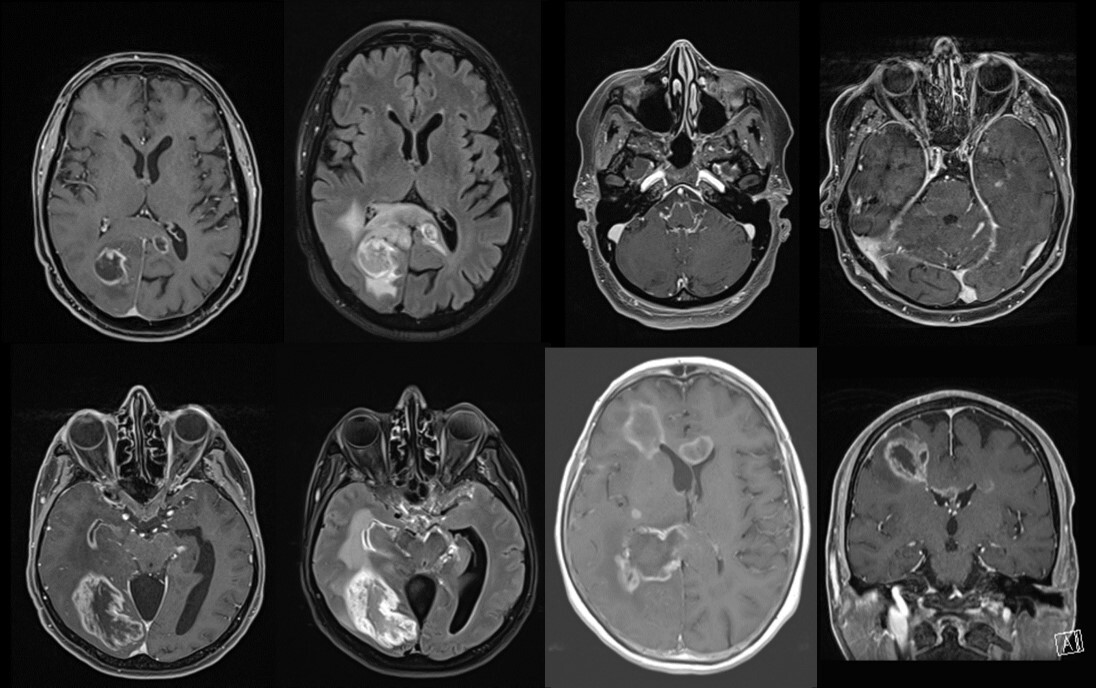
**

**Supplementary Fig. 1 a-d** Spread patterns in 2 different patients with mGBM. **a** (T1 with contrast) and **b** (FLAIR) multifocal glioblastoma with lesions in both hemispheres, pericallosal and periventricular spread (lesions within 1 cm of the ventricular system and the corpus callosum). **c** (T1 with contrast) and **d** (FLAIR) bilateral, subependymal and subarachnoidal spread (diffuse leptomeningeal contrast enhancement along the surface of the mesencephalon). **e** (T1 with contrast) **s**ubarachnoid spread: diffuse perimesencephalic enhancement. **f** (T1 with contrast) subarachnoid spread along the cerebellar fissures and two periventricular lesions (“periventricular spread”). **g** (T1 with contrast) and **h** (T1 with contrast) two different cases with multiple lesions: bilateral, periventricular, pericallosal and subependymal spread patterns.

h

g

a

b

c

dD

e

f
